# Physical, mental and behavioral health indicators in relation to academic performance in European boys and girls: the I.Family study

**DOI:** 10.1186/s12889-025-23578-3

**Published:** 2025-06-26

**Authors:** Qinyun Lin, Monica Hunsberger, Sofia Klingberg, Stefaan de Henauw, Antje Hebestreit, Fabio Lauria, Artur Mazur, Luis Moreno, Kurdiné Molnár Eszter Noémi, Iris Pigeot, Michael Tornaritis, Toomas Veidebaum, Lauren Lissner

**Affiliations:** 1 School of Public Health and Community Medicine, Institute of Medicine, Sahlgrenska Academy, University of Gothenburg, Guldhedsgatan 5A, Plan 3, Gothenburg, Sweden; 2 Department of Internal Medicine and Clinical Nutrition, University of Gothenburg, Gothenburg, Sweden; 3 Department of Public Health/Department of Movement and Sport Sciences, Faculty of Medicine and Health Sciences (UGENT), Ghent University, Ghent, Belgium; 4 Leibniz Institute for Prevention Research and Epidemiology – BIPS, Bremen, Germany; 5 National Research Council, Institute of Food Sciences (ISA-CNR), Unit of Epidemiology and Population Genetics, Avellino, Italy; 6 University of Rzeszów, Rzeszów, Poland; 7 University of Zaragoza, Zaragoza, Spain; 8 Department of Languages for Biomedical Purposes and Communication, Medical School, University of Pécs, Pécs, Hungary; 9 Research and Education Institute of Child Health, Nicosia, Cyprus; 10 National Institute for Health Development (NIHD), Tervise Arengu Instituut, Tallinn, Estonia

**Keywords:** Academic performance, Mathematics, Language, Adolescents, Physical fitness, Mental health, Sleep, Media use, Physical activity, Diet, Sex

## Abstract

**Background:**

Academic performance in children is associated with a range of health-related factors, including physical fitness, mental well-being, sleep, and behavioral patterns. While previous studies have examined these factors individually, fewer have assessed their independent associations with academic achievement while accounting for other relevant health indicators. This study uses data from the I.Family study to explore how physical, mental, sleep-related, and behavioral health indicators relate to academic achievement among European adolescents, considering each factor’s contribution while adjusting for the others.

**Methods:**

We used data from the 2013–2014 wave of the I.Family study to investigate eight health indicators: health related quality of life (HRQoL), body mass index (BMI), diet, media use, physical activity, sleep duration and quality, and stressful life events. Their associations with self-reported academic performance in mathematics and language were analyzed using binary logistic regression models, adjusting for confounders such as parents’ education, income, survey country and child’s age. We conducted separate analyses for girls and boys to capture associations that are specific to academic subject and sex.

**Results:**

A number of significant associations were found between several health indicators and academic performance. Higher HRQoL scores, reduced media time, and increased physical activity were linked to better academic performance in both mathematics and language for both boys and girls. Variation by sex and academic subjects were observed, with lower BMI, higher healthy diet scores and better sleep quality associated with better academic performance in language among girls. For mathematics, emotional, self-esteem, and family-related HRQoL were all significantly associated with higher performance for both boys and girls. In contrast, for language achievement, only family-related HRQoL was significant for both sexes.

**Conclusions:**

Our study underscores the need to consider both the importance of accounting for heterogeneity in sex and the differences between math and language academic subjects when investigating determinants of academic performance, setting the stage for further research on this topic to explore potential competing, synergistic, or time-dependent effects among these different health dimensions.

**Supplementary Information:**

The online version contains supplementary material available at 10.1186/s12889-025-23578-3.

## Background

The determinants of academic performance in adolescents are complex and encompass various multidimensional aspects. Previous empirical investigations have highlighted that these determinants may include chronic and acute health conditions [1, 2], physical fitness [3, 4], mental well-being [5–7], sleep duration and quality [8, 9], along with social conditions in families and schools [10, 11]. Research suggests that chronic diseases in adolescence can negatively influence academic pathways, limiting educational outcomes [1, 2]. Physical fitness has been found to positively impact cognitive function and attention, which are crucial for academic performance [3], while emotional well-being strongly predicts educational achievement and mediates the effect of poverty on educational outcomes [7]. Sleep deprivation may impair cognitive functions, reducing academic performance [8], while better sleep consistency is linked to better academic outcomes [9]. While previous studies have examined these determinants individually, fewer have assessed their independent associations with academic achievement while accounting for other relevant health indicators, given the dearth of epidemiological studies with data on school performance. Using data from the I.Family study, this study aims to investigate how physical, mental, sleep-related, and behavioral health indicators relate to academic achievement among European adolescents, considering each factor’s contribution while adjusting for the others.

Based on existing literature, health has always been viewed as a multifaceted construct, whether in the traditional definition of health by the World Health Organization (WHO) as “complete well-being,” or in the more recent trend to regard health as the “ability to adapt and self-manage” in the face of various challenges [12]. In both cases, health spans across dimensions of physical, mental, and social well-being, all of which are interconnected and cannot be fully understood in isolation. Each dimension influences and is influenced by the others, making it critical to adopt a comprehensive approach that integrates all these aspects. Accordingly, we hypothesize that academic performance is influenced by the joint effects of physical, mental, behavioral, and sleep-related health indicators. Additionally, behavioral factors such as media use and physical activity may impact academic performance directly or indirectly through physical and mental health indicators. For instance, interventions targeting physical activity could enhance academic performance by improving physical fitness [13, 14], mental well-being [15, 16] or sleep quality [17, 18]. At the same time, more engagement in physical activity might also directly influence academic performance by reducing hyperactivity and inattention, as well as fostering collaborative peer relationships [19, 20].

It is also crucial to consider the potential for heterogeneity in sex and the differences between mathematics and language subjects when investigating how health indicators impact academic performance. Research suggests that these effects may differ across sexes and academic subjects, such as mathematics and language. For example, a meta-analysis examining the effect of physical activity on academic performance found varying effect sizes depending on whether academic performance were measured as language-related skills, mathematics-related skills, or composite scores [21]. Additionally, studies show that girls typically achieve higher grades in school than boys [22, 23] especially in reading subjects [24], but also experience greater internal distress, more negative self-evaluations, and increased academic anxiety in school [25, 26], indicating possible sex differences in how mental well-being relates to academic performance [27].

Physical activity has also been found to have sex-specific effects; for example, active learning, which combines academic content with physical activity, benefits all boys but only low-achieving girls [28]. Furthermore, research has demonstrated significant yet small reciprocal relationship between BMI and math achievement for girls, but not for reading, with no such evidence observed for boys [29]. Sex differences may also exist in the impact of sleep on academic performance. A study of adolescents found that boys who slept longer than the age-specific recommendations had lower academic grades, whereas this association was not observed in girls, suggesting that excessive sleep may negatively impact boys’ academic performance while leaving girls unaffected [23].

Given our conceptualization that academic performance is jointly influenced by multiple health related factors, we leverage the I.Family data to explore how physical, mental, behavioral, and sleep-related health indicators relate to academic performance among adolescents, while accounting for heterogeneity in both sex- and academic subject. Below, we first describe the methodology, followed by the presentation of results and an in-depth discussion of the findings.

## Methods

### Data and study design

The I.Family study is based on a cohort from nine European countries: Belgium, Cyprus, Estonia, Germany, Hungary, Italy, Spain, Sweden and Poland [30]. This cohort was initially recruited from eight countries during the IDEFICS study from 2007 to 2008, when the children were two to ten years old [31]. The I.Family study subsequently expanded this cohort to include a new country (Poland), as well as including children’s siblings and parents, which allows for a more comprehensive analysis of family health dynamics. In all survey waves, data were gathered through physical examinations, including anthropometric measures, and self-administered questionnaires completed by adolescents aged 12 or older [32]. Questionnaires were identical across the nine countries, with slight adaptions for language and cultural contexts. A more detailed description about the cohort, including recruitment strategies and representativeness, can be found in Ahrens et al. [30, 31].

As described earlier, our theoretical conceptualization assumes that various health indicators may influence each other and jointly impact academic performance (e.g., screen time or sleep may impact academic performance indirectly through physical and mental health indicators). The present analysis is based on data from the third examination wave (I.Family, 2013–2014) when academic performance outcomes could first be reported by adolescents in each survey country. Although earlier-life exposure data is available in many of the original participants, the present sample size was optimized with data from siblings who were recruited at the third examination. Consequently, we opted for a cross-sectional design, positioning this study as an exploratory analysis of how different health indicators collectively associate with academic performance.

**Fig. 1:**
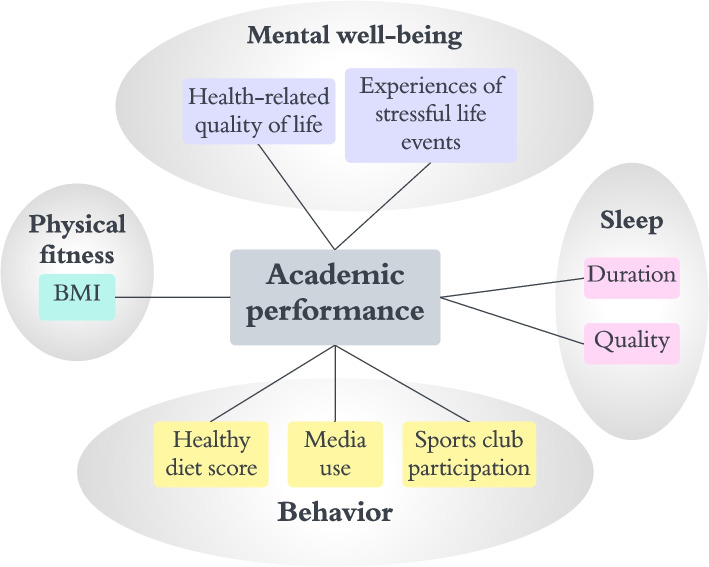
Analytical approach to study associations between academic performance and physical, mental, behavioral, and sleep-related health indicators

Figure 1 illustrates our *analytical approach*, in which we identified eight health indicators based on their relevance to physical, mental, behavioral and other sleep-related aspects of adolescent health, ensuring a comprehensive assessment of available data. These indicators include mental well-being (comprising the KINDL health-related quality of life [HRQoL] score and stressful life events), physical fitness (represented by BMI), sleep (with separate indicators for duration and quality), and behavioral indicators (including healthy diet score, media use, and sports club participation). Sleep is treated as a separate domain due to its dual role as both a physical and behavioral factor, influencing both daily functioning and overall well-being. This distinction allows for a more nuanced understanding of sleep’s unique contribution to academic performance.

Rather than modeling complex interdependences among these indicators (e.g., through path analysis), we use multivariable regression models to assess the effect of each health indicator while accounting for the presence of others, or more formally, conditional on other indicators. Given our study design and available data, this approach allows us to explore key associations while recognizing the need for further research to establish more intricate relationships. Adolescents aged 12 to 16.9 years with non-missing academic performance data were included in the analysis (*N* = 3,388).

The IDEFICS and I.Family study protocols adhered to the Declaration of Helsinki ethics principles for research that include human subjects. Participant inclusion required that parents give written informed consent for their children, and young children also gave verbal consent. From the age of 12 years onwards, children gave their own written informed consent in plain language. We followed the general data protection regulation (GDPR). Data are stored on a secure central server with password encryption, and samples are stored in secure biobanks. This study was coordinated by the Leibniz Institute for Prevention Research and Epidemiology – BIPS (Germany), and survey centers obtained ethics approval from respective local institutions in each country. Below, we provide detailed information on all the measures.

### Measures

#### Outcomes: academic performance

To measure academic performance, we used self-reported grades in mathematics and their own country’s native language, as these two subjects are most consistently graded across curricula in all participating countries. Grades were harmonized using the five-point European Credit Transfer System (ECTS) [33] to standardize them into five levels: excellent, good, satisfactory or mediocre, pass or sufficient, and fail or insufficient.

For this study, we dichotomized these levels into a binary indicator for each subject, categorizing grades as either higher performance (i.e., excellent and good) or all other levels. This resulted in one indicator per subject (mathematics and language) representing whether the participant reported higher performance in that subject. This decision was based on both theoretical and empirical considerations. Conceptually, the middle and lower performance categories may reflect shared risk factors for underachievement and combining them supports a clearer interpretation focused on academic success. Empirically, the number of participants in the lowest category (“fail or insufficient”) was small across subgroups (≤ 6%), limiting model stability. In follow-up sensitivity analyses, we also explored ordinal logistic models but found that the proportional odds assumption was significantly violated in all sex- and subject-stratified models, with extensive data sparsity and poor model fit. These findings reinforced the use of a dichotomized outcome to ensure robust and interpretable comparisons.

#### Predictors of interest: mental, physical, sleep-related, and behavioral health indicators

As described earlier, we identified eight indicators encompassing physical, mental, sleep-related, and behavioral aspects that may relate to self-reported academic performance (Fig. 1).

First, the mental well-being aspect include the KINDL HRQoL score and an assessment of stressful life events. The KINDL questionnaire, a widely used instrument for measuring health-related quality of life (HRQoL) in children and adolescents [34, 35], serves as the basis for the mental well-being aspect in this study. Since HRQoL captures individuals’ subjective perception of well-being, the KINDL score is considered a valid and meaningful indicator in this context. The questionnaire consists of 12 questions across four domains: emotional well-being, self-esteem, family, and social contacts (see Appendix for questions). Respondents were asked to report on how they had been feeling during the past week. Each domain was scored on a scale from 0 to 12, with an overall score ranging from 0 to 48, where higher scores indicate better well-being.

For stressful life events, participants reported whether they had experienced any of 13 potentially stressful events ever, such as parental divorce, the death of a parent, or the death of a family member (see Appendix for the complete list) [32]. We acknowledge that these events may have occurred at different points in time. However, previous research has shown that both long-term and recent stressful experiences contribute to mental well-being, with potential cumulative effects over time [36–38]. If they had experienced an event, they indicated the level of distress it caused, either “rather strongly” (score = 2) or “rather little” (score = 1). We then calculated a total score by summing these responses across all 13 events, yielding a possible range from 0 to 26, with higher scores indicating greater distress from these life events.

Second, body mass index (BMI) was used as an indicator of body composition, a key dimension of physical health. Trained personnel measured participants’ weight to the nearest 0.1 kg using a TANITA BC 420 MA scale and height to the nearest 0.1 cm using a SECA 225 stadiometer [30, 39]. Participants were measured early in the day wearing only light clothing. Age- and sex- specific BMI z-scores were then constructed based on an external standard population to standardize participants’ weight status [40].

Third, sleep indicators included nocturnal sleep duration and sleep quality. Participants reported their usual bedtime and wake-up time on both weekdays and on weekends [32]. We calculated nocturnal sleep duration as the average hours per night across both weekdays and weekends. For sleep quality, participants evaluated their sleep during the past month on a four-point scale: very good, fairly good, fairly bad, and very bad [32]. We then created a binary indicator, with “good” sleep quality defined as either very or fairly good (1) and “not good” as fairly bad or very bad (0).

Lastly, behavioral indicators include a healthy diet adherence score, media use, and involvement in physical activities in leisure time. The healthy diet adherence score was derived from frequency data in the validated Food Frequency Questionnaire, where participants reported how many times they had consumed specific food items in the past month. The score comprised five sub-scores reflecting adherence to nutrition guidelines on consumption for fruit and vegetables, fish, whole grains, fat quantity, and sugar. The overall score ranged from 0 to 50, with higher values indicating better adherence to these guidelines [32, 39]. This questionnaire has been previously assessed against 24-h dietary recall data in validation studies, demonstrating its reliability in estimating dietary intake patterns [32].

Sedentary media use was measured by the self-reported number of hours spent with audiovisual media, including personal computers, game consoles, and televisions, over an entire week. Participants were asked, “How long do you usually watch TV and/or video/DVD per day?” and “How long do you usually sit at a computer/game console per day?” They provide answers separately for weekdays and weekends. We then calculated the total hours spent on these activities over the entire week.

Sports club participation was used as a proxy for physical activity. Participants were first asked whether they were members of a sports club. If they answered “no,” their weekly time spent engaging in sports at a sports club was recorded as zero. If they answered “yes,” they were then asked, “How much time do you usually spend doing sport in a sports club per week?”. This measure was used as a proxy for their level of organized sports in leisure time (leisure time physical activity, LTPA). While objective accelerometry data was not available for all countries, and was missing in a substantial number of cases, sports club participation was considered a valid, subjective proxy for physical activity. Among participants with available data on moderate-to-vigorous physical activity (MVPA), a small but highly significant correlation was found between sports club participation and MVPA (0.19, *p* < 0.001). Given its availability of data across all countries, we used sports club participation as a comprehensive measure to preserve sample size and ensure consistency across countries in the analysis.

#### Control variables/covariates

In all multivariable models, we included parents’ education and income, as well as adolescents’ age, as covariates. The parent completing the questionnaire reported both their own and their partner’s educational attainment. The highest level of education between the two was categorized according to International Standard Classification of Education (ISCED) [41] to ensure comparability across countries. Parental education was further categorized into three levels: low, medium, and high. Reported family income was standardized across participating countries by linking each household’s income to the average country-specific net household income. This income was then categorized into four levels: low/medium, medium, medium/high, and high [31].

### Statistical analysis

We started with descriptive analyses, comparing academic performance and all mental, physical, sleep-related, and behavioral indicators with stratification on sex. Statistical comparisons were conducted using Wilcoxon rank sum tests and Pearson’s chi-square tests.

To examine the associations between these indicators and academic performance, we employed binary logistic regression models. To account for potential country-specific effects, we used logistic regression models with country fixed effects, with eight dummy variables to represent the nine countries. In all models, parents’ education, income, and adolescents’ age were included as covariates. Additionally, to account for potential sex-related heterogeneity and difference between mathematics and language subjects, we conducted separate analyses for girls and boys, as well as for language and mathematics subjects. This approach allows us to explore how the identified factors may influence academic performance differently across sex and academic subject categories, providing a more nuanced understanding of these associations.

Given the potential collinearity among the eight mental, physical, sleep, and behavioral indicators, we started our multivariable analyses with optimized sample sizes by including each indicator in a separate model, with parental education, income and adolescents’ age as covariates. We then compared these single-indicator models to models including all eight indicators, using pairwise deletion. Since the results were similar, we report the findings from the models with all indicators in the results section, while the single-indicator models are presented in the Appendix as sensitivity analyses. As an additional check for multicollinearity, we calculated Spearman correlation among all eight health indicators, and we found that all correlations were below 0.19.

For the models with all indicators, we carried out further analyses to assess the effect of each specific dimension of the HRQoL score (i.e., emotional well-being, self-esteem, family, and social contacts). Again, considering the collinearity among these dimensions, we assessed each dimension separately, with each model including one dimension of the HRQoL score and the other seven health indicators as covariates. Similarly, stressful life events were initially analyzed as a composite score, summing the total scores of thirteen events. However, given unexpected findings in the primary analysis, we conducted additional exploratory analyses to examine the individual events contributing to the observed results.

We used pairwise deletion to handle missing data throughout the analyses, but multiple imputation was also performed as part of sensitivity analyses. Additional sensitivity analyses were conducted to assess the robustness of our findings concerning model specification (i.e., treating country as random effects rather than fixed effects). We also compared the single-indicator models with the models including all indicators. Furthermore, we examined the consistency of results when academic performance outcomes were defined using a reverse dichotomization of the original outcome. We also explored ordinal logistic regression using the original five-category outcome, but all sex- and subject-stratified models violated the proportional odds assumption and exhibited sparse data structures, reinforcing the choice of binary logistic regression.

All statistical tests were two-sided with a significance level set at 5%. In binary logistic regression analyses, we reported the results of all pre-planned comparisons with *p*-values without adjustment for multiplicity. Thus, marginally significant results must be interpreted with caution. All statistical analyses were performed using the R software environment (version 4.4.1) and STATA (version 18.0).

## Results

In Table 1, we present the distribution of mathematics and language grades for boys and girls using the original five-level scale. Figure S1 in the Appendix shows the proportion of boys and girls who achieve the highest two levels in mathematics and language, respectively. Both Table 1 and Figure S1 highlight statistically significant differences between boys and girls in both mathematics and language subjects. In mathematics, 46.5% of boys reported achieving the highest two levels (95% CI: [44.0%, 48.9%]), compared to 51.7% of girls (95% CI: [49.3%, 54.1%]). The difference is even more pronounced in language, where 45.2% of boys (95% CI: [42.80%, 47.7%]) and 58.4% of girls (95% CI: [56.1%, 60.8%]) reached the highest two levels. Thus, girls outperformed boys by approximately 5 percentage points in mathematics and 13 percentage points in language. Consistently, the chi-square tests based on the five-level scale confirmed statistically significant differences between boys and girls in both mathematics (*p* = 0.015) and language (*p* < 0.001).

**Table 1:** Academic outcomes (math and language grades) by sex groups (five-level scale)

**Variable**	**Boys** *N* = 1,658	**Girls** *N* = 1,730	***P*-value**
Math (5 levels)			0.015
Excellent	317 (19%)	381 (22%)	
Good	441 (27%)	504 (29%)	
Satisfactory or mediocre	532 (33%)	535 (31%)	
Pass or sufficient	244 (15%)	216 (13%)	
Fail or insufficient	97 (6%)	76 (4%)	
Language (5 levels)			< 0.001
Excellent	276 (17%)	469 (27%)	
Good	468 (28%)	536 (31%)	
Satisfactory or mediocre	563 (34%)	523 (30%)	
Pass or sufficient	251 (15%)	139 (8%)	
Fail or insufficient	87 (5%)	53 (3%)	

Frequency (percentage) was provided for each variable. *P*-values were calculated to compare boys and girls based on Pearson’s Chi-squared tests

In Table 2, we present summary statistics, describing sex differences in all independent variables. Boys and girls are similar in age, parental education, and family income. However, several indicators show statistically significant sex differences. These include higher HRQoL scores in boys (specifically in emotional well-being and self-esteem), higher BMI z-scores, more media use, more hours spent in sports clubs, longer nocturnal sleep, and better sleep quality.

**Table 2:** Summary statistics of predictors of interest and covariates in this study by sex groups

**Variable**	**Overall** *N* = 3,388	**Boys** *N* = 1,658	**Girls** *N* = 1,730
Age	13.60 (13.00, 14.10)	13.50 (13.00, 14.10)	13.60 (13.00, 14.10)
Parental education			
Low	203 (6%)	97 (6%)	106 (6%)
Medium	1,442 (45%)	711 (45%)	731 (44%)
High	1,583 (49%)	765 (49%)	818 (49%)
Family income			
Low/medium	842 (31%)	440 (33%)	402 (29%)
Medium	999 (36%)	483 (36%)	516 (37%)
Medium/high	323 (12%)	147 (11%)	176 (13%)
High	581 (21%)	272 (20%)	309 (22%)
HRQoL (overall)^a^	39.00 (35.00, 42.00)	39.00 (35.00, 42.00)	38.00 (34.00, 42.00)
HRQoL (Emotional)^a^	9.00 (8.00, 11.00)	10.00 (9.00, 11.00)	9.00 (8.00, 10.00)
HRQoL (Self-Esteem)^a^	10.00 (8.00, 11.00)	10.00 (8.00, 11.00)	9.00 (8.00, 11.00)
HRQoL (Family)	10.00 (9.00, 11.00)	10.00 (9.00, 11.00)	10.00 (9.00, 11.00)
HRQoL (Friends)^a^	10.00 (9.00, 11.00)	10.00 (9.00, 11.00)	10.00 (9.00, 11.00)
Stressful life events	2.00 (0.00, 3.00)	2.00 (0.00, 3.00)	2.00 (0.00, 3.00)
BMI z-score^a^	0.58 (−0.16, 1.37)	0.69 (−0.11, 1.53)	0.48 (−0.21, 1.25)
Healthy diet score^a^	20.00 (15.00, 25.00)	20.00 (15.00, 24.00)	20.00 (15.00, 25.00)
Weekly media use (hours)^a^	17.25 (10.50, 25.75)	21.00 (13.50, 30.00)	14.25 (8.50, 22.25)
Sports club (hours)^a^	1.50 (0.00, 4.00)	2.00 (0.00, 4.50)	1.00 (0.00, 3.23)
Average nightly sleep duration (hours)^a^	8.63 (8.06, 9.29)	8.69 (8.14, 9.33)	8.57 (8.00, 9.29)
Sleep quality^a^			
Good	3,031 (91%)	1,510 (92%)	1,521 (89%)
Bad	308 (9%)	123 (8%)	185 (11%)

Median (IQR) was provided for continuous variables and frequency (percentage) was provided for categorical variables. Table S1 in the Appendix details missing information for all variables. Missing data are labeled as “Unknown” in Table S1

^a^Indicates statistically significant differences between boys and girls at 5% significance level based on Wilcoxon rank sum tests for continuous variables and Pearson’s Chi-squared tests for categorical variables

In Table 3, we present the results from multivariable logistic regression models that include all key indicators. Each column represents a model predicting high performance in mathematics and language for boys and girls, respectively. For better visualization of the results, see Figure S2 in the Appendix which provides forest plots with all indicators of interest. Several consistent trends emerge for predicting high achievement in mathematics: higher overall HRQoL scores (OR [95% CI]: boys 1.07 [1.04, 1.10], *p* < 0.001; girls 1.06 [1.03, 1.09], *p* < 0.001), less media use (boys 0.98 [0.97, 0.997], *p* = 0.013; girls 0.99 [0.97, 0.999], *p* = 0.039) and more sports club hours (boys 1.08 [1.03, 1.13], *p* < 0.001; girls 1.13 [1.07, 1.19], *p* < 0.001) are significantly related with higher odds of achieving high performance in mathematics. Unexpectedly, more stressful life events are associated with increased odds of high performance in mathematics for boys (1.07 [1.01, 1.14], *p* = 0.026). Additional exploratory analyses revealed that this association is mainly driven by the event “Death of a grandparent or other family member” (1.43, [1.16, 1.76], *p* = 0.001).

**Table 3:** Fully adjusted multivariable logistic regression models predicting high math and language grades by sex groups

**Characteristic**	**Boy (High Math grade)**			**Girl (High Math grade)**			**Boy (High Language grade)**			**Girl (High Language grade)**		
	**OR**	**95% CI**	***P*-value**	**OR**	**95% CI**	***P*-value**	**OR**	**95% CI**	***P*-value**	**OR**	**95% CI**	***P*-value**
Age	0.78	0.67, 0.91	0.001	0.75	0.64, 0.88	< 0.001	0.81	0.69, 0.96	0.012	0.85	0.71, 1.00	0.052
Parental education	reference: low											
Medium	4.12	1.48, 14.8	0.014	1.46	0.67, 3.43	0.4	1.87	0.73, 5.39	0.2	1.30	0.59, 3.03	0.5
High	6.18	2.18, 22.6	0.002	1.98	0.88, 4.77	0.11	2.61	1.00, 7.71	0.063	2.04	0.88, 4.90	0.10
Family income	reference: low/medium											
Medium	1.34	0.93, 1.93	0.12	1.23	0.84, 1.80	0.3	1.41	0.96, 2.07	0.082	1.46	0.97, 2.18	0.068
Medium/high	2.74	1.59, 4.78	< 0.001	1.16	0.69, 1.94	0.6	2.34	1.32, 4.21	0.004	1.59	0.90, 2.83	0.11
High	2.20	1.36, 3.56	0.001	2.25	1.41, 3.60	< 0.001	2.57	1.53, 4.35	< 0.001	1.78	1.06, 2.97	0.029
HRQoL (overall)	1.07	1.04, 1.10	< 0.001	1.06	1.03, 1.09	< 0.001	1.04	1.01, 1.07	0.013	1.03	1.002, 1.06	0.033
Stressful life events	1.07	1.01, 1.14	0.026	0.96	0.91, 1.02	0.2	1.00	0.94, 1.07	> 0.9	1.00	0.93, 1.06	> 0.9
BMI z-score	0.90	0.79, 1.03	0.11	0.94	0.82, 1.08	0.4	0.96	0.83, 1.10	0.6	0.78	0.67, 0.90	0.001
Healthy diet score	1.00	0.98, 1.02	0.7	1.00	0.99, 1.02	0.7	1.02	0.99, 1.04	0.2	1.03	1.01, 1.05	0.004
Weekly media use (hours)	0.98	0.97, 0.997	0.013	0.99	0.97, 0.999	0.039	0.97	0.96, 0.99	< 0.001	0.97	0.96, 0.99	< 0.001
Sports club (hours)	1.08	1.03, 1.13	< 0.001	1.13	1.07, 1.19	< 0.001	1.06	1.01, 1.11	0.029	1.08	1.02, 1.15	0.008
Average nightly sleep duration (hours)	1.09	0.93, 1.27	0.3	0.87	0.75, 1.01	0.073	1.08	0.92, 1.27	0.4	0.88	0.75, 1.03	0.12
Sleep quality	reference: good											
Bad	0.75	0.43, 1.31	0.3	0.87	0.54, 1.40	0.6	0.73	0.40, 1.31	0.3	0.57	0.34, 0.96	0.034
Observations (N)	1051			1132			1050			1140		

All models include country fixed effects (9 countries, represented by 8 dummy variables), but these are omitted from the table as they are not of primary interest. Odds ratio (OR) and 95% CI were provided in the table. “Math” and “Language” in column headers refer to the outcome variable, indicating the likelihood of achieving a high grade in the respective subject. We reported the results of all pre-planned comparisons with *p*-values. This is a complete-case analysis, only including participants with all independent variables and at least one academic subject grade available

For language achievement, higher overall HRQoL scores (boys: 1.04 [1.01, 1.07], *p* = 0.013; girls: 1.03 [1.002, 1.06], *p* = 0.033), less media use (boys: 0.97 [0.96, 0.99], *p* < 0.001; girls: 0.97 [0.96, 0.99], *p* < 0.001), and more hours spent in sports clubs (boys: 1.06 [1.01, 1.11], *p* = 0.029; girls: 1.08 [1.02, 1.15], *p* = 0.008) are significant for both boys and girls. Additionally, three health indicators are significantly associated with high language achievement in girls but not in boys: lower BMI z-scores (0.78 [0.67, 0.90], *p* = 0.001), higher healthy diet scores (1.03 [1.01, 1.05], *p* = 0.004) and better sleep quality (0.57, [0.34, 0.96], *p* = 0.034; with “good” sleep quality as the reference category).

**Fig. 2:**
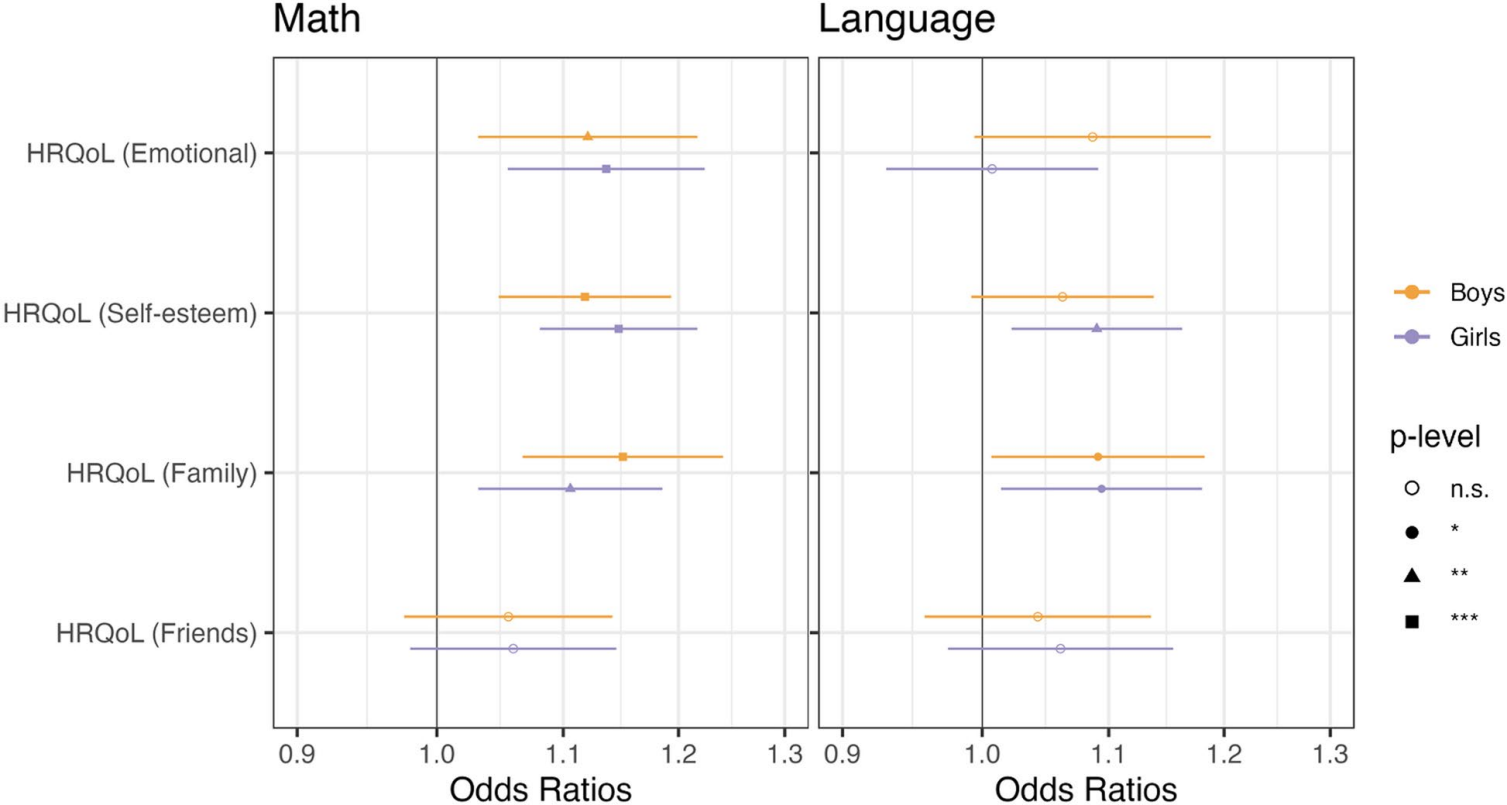
Forest plots for specific dimensions of HRQoL scores in the fully adjusted multivariable logistic regression models predicting math and language grades by sex groups. Note: The odds ratios and 95% CI were reported in this Figure. The results were from separate models: four models predicting math high achievement for girls, four models predicting math high achievement for boys, four models predicting language high achievement for girls and another four models predicting language high achievement for boys. Each model focuses on a single HRQoL dimension. All models include the other seven key health predictors (i.e., stressful life events, BMI z-score, healthy diet score, media use, hours spent in sports clubs, nocturnal sleep duration and sleep duration) and covariates (i.e., age, parental education, family income and country fixed effects). *** *p* < 0.001, ** *p* < 0.01, * *p* < 0.05, n.s. indicates not significant at 5% level

We further examined the association between each specific HRQoL dimension and academic performance in mathematics and language, with results presented in Fig. 2. Consistent with the overall HRQoL pattern, higher scores in specific well-being dimensions are generally associated with improved odds of high performance, although these associations vary by dimension, sex and academic subject.

For both boys and girls, higher odds of high achievement in mathematics are significantly associated with better emotional HRQoL (boys: 1.12 [1.03, 1.22], *p* = 0.007; girls: 1.14 [1.06, 1.22], *p* < 0.001), higher self-esteem (boys: 1.12 [1.05, 1.19], *p* < 0.001; girls: 1.15 [1.08, 1.22], *p* < 0.001), and greater family-related HRQoL (boys: 1.15 [1.07, 1.24], *p* < 0.001; girls (1.11 [1.03, 1.19], *p* = 0.005).

In contrast, the associations between HRQoL and language achievement appear weaker and more variable by sex. For girls, both self-esteem and family-related HRQoL remained significant (self-esteem: 1.09 [1.02, 1.16], *p* = 0.008; family-related: 1.09 [1.01, 1.18]; *p* = 0.020). For boys, however, only family-related HRQoL (1.09 [1.01, 1.18]; *p* = 0.03) is significant at 5% level, while emotional HRQoL (1.09 [0.995, 1.19], *p* = 0.067) and self-esteem (1.06 [0.992, 1.14], *p* = 0.084) show a trend approaching significance. Notably, friends-related HRQoL is not significantly associated with either math or language achievement for either sex group.

Findings from the sensitivity analyses are presented in the Appendix. Table S2 present results for single-predictor models, Table S3 show results when countries were modeled as random effects (i.e., multilevel models), and Table S4 provide results when multiple imputation was performed for missing data instead of pairwise deletion. Across these analyses, the results remained highly consistent. Additionally, we conducted an alternative analysis in which academic performance was dichotomized in the reverse direction – classifying “Pass or sufficient” and “Fail or insufficient” as poor outcomes (1), compared to higher grades (0). As shown in Table S5, this approach yielded results that were broadly consistent with the original analysis: improved well-being, a higher healthy diet score, reduced media use, and more club hours were associated with lower odds of achieving low academic outcomes, though the effects were weaker. Notably, the sex- and academic subject-specific effects may differ depending on whether the focus is on predicting higher or lower academic achievement, as these distinctions may reflect different underlying mechanisms and contributing factors.

## Discussion

As hypothesized, our study reveals significant and consistent associations between several indicators and adolescents’ academic performance. Higher well-being scores, reduced media usage, and increased physical activity demonstrate statistically significant associations with improved academic performance in both mathematics and language subjects for both girls and boys. However, other indicators, such as stressful life events, BMI, healthy diet score, and sleep, exhibit different effects across sex and between mathematics and language. In the following discussion, we first discuss the three indicators with consistent effects before exploring the more nuanced, sex- and academic subject-specific findings.

First, our study found that higher overall HRQoL scores are consistently associated with better performance in both mathematics and language for boys and girls. This aligns with existing literature confirming the positive relationship between subjective well-being and academic performance [27, 42]. To further explore these associations, we examined specific dimensions of HRQoL, revealing a slightly stronger mathematics-specific effect, where emotional, self-esteem, and family-related well-being are all significantly related to high achievement for both boys and girls. Among these, self-esteem and family-related HRQoL were also significantly linked to language achievement in girls, while for boys, only family-related well-being remained significant. In contrast, friends-related HRQoL is not significantly associated with academic performance in either subject or sex group. It is possible that peer-related well-being is less directly tied to academic achievement compared to emotional or family domains, or that its influence is more context-dependent – such as whether friendships promote academic engagement or distraction – and not well captured by the measure used in this study.

One possible explanation for the stronger effect of self-esteem and emotional well-being on mathematics achievement is that these factors may enhance confidence and reduce anxiety, both of which are particularly relevant for mathematics performance compared to language [43, 44]. However, it is also important to acknowledge potential bi-directional associations, as prior academic success or struggles can shape students’ subjective well-being over time. For example, low self-esteem may stem from previous negative experiences with learning mathematics [45, 46]. Additionally, Bortes et al.’s [42] found that high academic achievement (not specifically mathematics) can be associated with declines in subjective well-being over time, with these effects observed predominantly among girls. While our study provides a cross-sectional snapshot, longitudinal research would be valuable for disentangling these complex, reciprocal effects and examining potential differences by sex and between mathematics and language subjects in greater detail.

Second, less media use consistently predicts better academic performance for both boys and girls, across mathematics and language subjects. This finding aligns with previous literature, such as Adelantado-Renau et al. [47], who reviewed 58 cross-sectional studies and confirmed the inverse association between screen-based activities and academic performance, noting stronger effects in adolescents compared to children. While our data does not differentiate the type or context of media use, it supports the existing evidence of its negative impact on academic performance for both sexes. Notably, other studies have reported interesting sex differences. For instance, Martinez-Gomez et al. [48] found no association for boys, whereas our study observed a negative effect on both sexes. Other research indicates that boys and girls use media differently – boys often engage in intense video gaming and girls in cell phone use [49]. Additionally, emerging research suggests that different types and contexts of screen-based activities—such as social media, video gaming, and passive television watching—as well as whether these activities are engaged alone or with parents, may have distinct effects on academic performance [47, 50]. This underscores the need for further research to explore how the quantity, content, and context of media use may affect girls and boys differently and tailor interventions accordingly.

Another behavioral indicator in our study, physical activity, measured by hours spent in organized sports, also shows consistent beneficial effects on both mathematics and language outcomes for both sex groups. These findings align with existing literature on positive effects of physical exercise on academic performance [21, 51–53]. Notably, review studies suggest that evidence is stronger for beneficial effects of physical activities on mathematics performance [21, 53], a trend also confirmed by our results (e.g., mathematics shows a highly significant effect with *p* < 0.001 for both boys and girls).

The healthy diet adherence score, as the final behavioral indicator, did not show any significant associations with academic performance for boys in our study. However, it displayed a significant association for girls regarding language performance (i.e., higher healthy diet score is associated with higher odds of achieving high performance). Interestingly, BMI was also significantly associated with girls’ language achievement, with a consistent pattern that lower BMI was related to better language performance. This finding contributes to the mixed literature on the relationship between obesity, nutrition, and cognitive and academic performance [54]. Our study provides insights into these mixed findings: the association between BMI (and the healthy diet score) and academic performance was observed exclusively for girls and specifically for language grades. This association persisted even after controlling for parents’ education, income, and other health indicators, such as physical activity and mental well-being. One possible explanation is that higher BMI may impede girls’ social-emotional development, such as forming collaborative peer relationships, which in turn could negatively affect cognitive abilities crucial for the language subject (e.g., comprehension and verbal reasoning). This aligns with other studies suggesting that overweight status is a significant risk factor for girls’ academic performance but not necessarily for boys [55, 56]. Such academic subject- and sex- specificity may help explain the insufficient evidence in recent research involving the direct link between obesity and academic performance [57].

Another notable sex- and academic subject-specific effect is the association between stressful life events and mathematics achievement among boys. Unexpectedly, our study showed more stressful life event experiences are associated with greater odds of achieving high performance in mathematics. To better understand this association, we explored specific events and found that it was primarily driven by the loss of a grandparent or another family member. While much of the existing literature emphasizes the negative impact of stressful life events [36–38], some studies suggest that exposure to adverse life events may foster subsequent resilience, ultimately benefiting cognitive functioning and mental health [58]. It is also possible that engaging in mathematics serves as a coping mechanism, helping boys distract themselves from stressors. Research suggests that boys may be more likely than girls to adopt avoidance-based coping strategies [59]. Compared to language, mathematics may offer a more effective form of distraction due to its procedure nature and lower emotional engagement. However, our study remains exploratory, further research is needed to better understand these dynamics and their implications.

Lastly, sleep quality is significantly associated with higher language achievement among girls, while sleep duration showed no significant associations in either the single-predictor (Table S2) or fully adjusted models. This finding aligns with a recent systematic review on sleep and academic performance suggesting that sleep quality may be a stronger predictor of academic performance than sleep duration [60]. However, the observed sex- and academic subject-specific effect of sleep quality remains difficult to explain, as prior research presents a complex picture of how sleep affects academic performance. For example, one study suggests that sleep duration primarily influences mathematics performance among girls [61], while chronotype effects may be more pronounced in boys [62]. One possible explanation is that sleep quality is closely linked to stress regulation and emotional well-being. Poor sleep quality has been associated with heightened stress and anxiety [63–65], and girls were known to be more prone to internal distress than boys [25]. Although mathematics performance is typically more sensitive to anxiety, the specific association between sleep quality and language achievement in girls suggests that sleep disturbances may interfere with cognitive processes such as verbal fluency, memory consolidation, and executive functioning—skills more directly involved in language-based tasks. However, we also want to note that it is possible that the effects of sleep operate indirectly through other health indicators included in our models, such as mental well-being and physical fitness, which may differentially impact language and mathematics outcomes. Future research, particularly longitudinal studies with more comprehensive measures of sleep, would help clarify these mechanisms and further explore the nuanced relationships between sleep, well-being, and academic performance.

## Limitations and strengths

This study has a number of strengths including the highly standardized protocols that were adapted in nine European countries. Another strength is that all of our main findings are adjusted for the family’s socioeconomic status, which is a known determinant of low academic performance. However, our findings are subject to limitations.

First, because this is a cross-sectional study, we cannot make causal inferences about the direction or nature of these relationships. Longitudinal studies with pretest measures of academic performance would be valuable to better understand causal dynamics and changes over time. However, this is not possible due to a challenge in the IDEFICS/I.Family cohort: younger children are not given school grades in some countries.

Second, our study relies on self-reported data for academic performance and some health indicators, such as media use and hours spent in organized sports. These measures could thus be subject to recall bias and social desirability bias, as respondents may not accurately remember or may misrepresent their conditions or behaviors. Additionally, the media use measure in our study may not fully represent current patterns, as it does not account for the increased use of social media and mobile in recent years.

Third, while BMI is widely used as a proxy for body composition, it is not a direct measure of body fatness, and cannot capture the complexity of body composition. Similarly, we used participation in organized sports clubs as a proxy for physical activity, which does not fully reflect the range or intensity of physical activity levels. Future studies could benefit from more precise measurements of these variables.

Fourth, we dichotomized academic performance into a binary outcome (higher vs. lower performance) rather than using the full five-category scale. This decision was based on both conceptual and statistical considerations: the lowest performance group (“fail or insufficient”) was small across subgroups, and ordinal logistic models consistently violated the proportional odds assumption and exhibited data sparsity. While dichotomization may reduce granularity, our robustness checks, including reverse dichotomization, support the consistency of the findings across outcome specifications.

## Conclusions

Our findings offer valuable insights into how various physical, mental, behavioral, and sleep health indicators are associated with academic performance in adolescents. While our study does not model complex interdependencies among these indicators, our multivariable regression approach allows us to estimate the association of each health indicator with academic performance, accounting for the presence of other indicators. A key result is the significant sex differences in these associations, particularly the inverse relationship between BMI and language performance, which was observed only in girls. Notably, media use was strongly associated with both mathematics and language outcomes for boys and girls, and engagement in organized sports was particularly associated with mathematics performance. Our study underscores the need to consider both the importance of accounting for heterogeneity in sex and the differences between math and language academic subjects when investigating determinants of academic performance, setting the stage for further research on this topic to explore potential competing, synergistic, or time-dependent effects among these different health dimensions.

## Supplementary Information

Supplementary Material 1.

## Data Availability

The data analysed in the current study are available from the corresponding author upon reasonable request, and with permission from the IDEFICS/I.Family consortium.

## Abbreviations

BMI: Body mass index
ECTS: European Credit Transfer System
LTPA: Leisure time physical activity
ISCED: International Standard Classification of Education
HRQoL: Health-related quality of life
