# Supplementary material for: Physical, mental and behavioral health indicators in relation to academic performance in European boys and girls: the I.Family study
Source: BMC Public Health. 2025 Jun 26;25:2176. doi: 10.1186/s12889-025-23578-3 (PMC12199524; doi:10.1186/s12889-025-23578-3)
Supplement: Supplementary file 1 — Supplementary Material 1. [file 12889_2025_23578_MOESM1_ESM.docx]

**Appendix**

**Table S1.** Summary statistics of all variables with missing information included.

| **Variable** | **Overall** N = 3,388 | **Boys** N = 1,658 | **Girls** N = 1,730 |
| --- | --- | --- | --- |
| **Age** | 13.60 (13.00, 14.10) | 13.50 (13.00, 14.10) | 13.60 (13.00, 14.10) |
| **Parental education** |  |  |  |
| low | 203 (6%) | 97 (6%) | 106 (6%) |
| medium | 1,442 (45%) | 711 (45%) | 731 (44%) |
| high | 1,583 (49%) | 765 (49%) | 818 (49%) |
| Unknown | 160 | 85 | 75 |
| **Family income** |  |  |  |
| low/medium | 842 (31%) | 440 (33%) | 402 (29%) |
| medium | 999 (36%) | 483 (36%) | 516 (37%) |
| medium/high | 323 (12%) | 147 (11%) | 176 (13%) |
| high | 581 (21%) | 272 (20%) | 309 (22%) |
| Unknown | 643 | 316 | 327 |
| **HRQoL (overall)** | 39.00 (35.00, 42.00) | 39.00 (35.00, 42.00) | 38.00 (34.00, 42.00) |
| Unknown | 195 | 99 | 96 |
| **HRQoL (Emotional)** | 9.00 (8.00, 11.00) | 10.00 (9.00, 11.00) | 9.00 (8.00, 10.00) |
| Unknown | 75 | 45 | 30 |
| **HRQoL (Self-Esteem)** | 10.00 (8.00, 11.00) | 10.00 (8.00, 11.00) | 9.00 (8.00, 11.00) |
| Unknown | 83 | 39 | 44 |
| **HRQoL (Family)** | 10.00 (9.00, 11.00) | 10.00 (9.00, 11.00) | 10.00 (9.00, 11.00) |
| Unknown | 109 | 51 | 58 |
| **HRQoL (Friends)** | 10.00 (9.00, 11.00) | 10.00 (9.00, 11.00) | 10.00 (9.00, 11.00) |
| Unknown | 76 | 35 | 41 |
| **Stressful life events** | 2.00 (0.00, 3.00) | 2.00 (0.00, 3.00) | 2.00 (0.00, 3.00) |
| **BMI z-score** | 0.58 (-0.16, 1.37) | 0.69 (-0.11, 1.53) | 0.48 (-0.21, 1.25) |
| Unknown | 12 | 6 | 6 |
| **Healthy diet score** | 20.00 (15.00, 25.00) | 20.00 (15.00, 24.00) | 20.00 (15.00, 25.00) |
| Unknown | 56 | 32 | 24 |
| **Media use (hours)** | 17.25 (10.50, 25.75) | 21.00 (13.50, 30.00) | 14.25 (8.50, 22.25) |
| Unknown | 177 | 96 | 81 |
| **Weekly sports club (hours)** | 3.50 (2.00, 6.00) | 4.00 (2.00, 6.00) | 3.00 (2.00, 5.50) |
| Unknown | 52 | 25 | 27 |
| **Average nightly sleep duration (hours)** | 8.63 (8.06, 9.29) | 8.69 (8.14, 9.33) | 8.57 (8.00, 9.29) |
| Unknown | 199 | 98 | 101 |
| **Sleep quality** |  |  |  |
| good | 3,031 (91%) | 1,510 (92%) | 1,521 (89%) |
| bad | 308 (9%) | 123 (8%) | 185 (11%) |
| Unknown | 49 | 25 | 24 |

***Note***. Median (IQR) was provided for continuous variables and frequency (percentage) was provided for categorical variables.

**Table S2:** Compare with single predictor models.

| Predictor | Boy (Math) | Girl (Math) | Boy (Language) | Girl (Language) |
| --- | --- | --- | --- | --- |
| HRQoL (overall) | 1.07 [1.04, 1.10], < .001 | 1.06 [1.04, 1.09], < .001 | 1.05 [1.02, 1.08] < .001 | 1.05 [1.02, 1.08] < .001 |
| Stressful life events | 1.05 [0.999, 1.11] 0.054 | 0.95 [0.90, 1.00]  0.067 | 0.995 [0.94, 1.05]  0.884 | 0.98 [0.92, 1.03]  0.409 |
| BMI z-score | 0.88 [0.78, 0.98]  0.025 | 0.90 [0.80, 1.02]  0.100 | 0.93 [0.82, 1.05]  0.247 | 0.78 [0.69, 0.89]  < .001 |
| Healthy diet score | 1.01 [0.99, 1.03]  0.288 | 1.02 [0.999, 1.03]  0.066 | 1.03 [1.01, 1.05]  0.005 | 1.03 [1.01, 1.05]  0.001 |
| Weekly media use (hours) | 0.99 [0.98, 0.997] 0.013 | 0.98 [0.97, 0.99]  0.001 | 0.98 [0.96, 0.99] < .001 | 0.96 [0.95, 0.98]  < .001 |
| Sports club (hours) | 1.09 [1.04, 1.13]  < .001 | 1.14 [1.09, 1.20]  < .001 | 1.07 [1.02, 1.12]  0.002 | 1.09 [1.04, 1.15]  .001 |
| Average nightly sleep duration (hours) | 1.10 [0.97, 1.26]  0.143 | 0.94 [0.82, 1.08]  0.378 | 1.14 [0.99, 1.31]  0.077 | 0.98 [0.85, 1.13]  0.753 |
| Sleep quality (good is reference) | 0.65 [0.40, 1.03]  0.069 | 0.73 [0.48, 1.10]  0.129 | 0.73 [0.45, 1.20]  0.214 | 0.58 [0.37, 0.90]  0.015 |

***Note***. Odds ratio (OR) and 95% CI were provided in this Table. The results were from separate models: eight models predicting math high achievement for girls, eight models predicting math high achievement for boys, eight models predicting language high achievement for girls and another eight models predicting language high achievement for boys. Each model focuses on a single indicator. All models include covariates (i.e., age, parental education, family income) and country fixed effects (9 countries, represented by 8 dummy variables).

**Table S3**. Fully adjusted multivariable logistic regression models predicting math and language grades by sex groups, with countries modeled as random effects (i.e., multilevel model).

|  | **Boy (Math)** | | | **Girl (Math)** | | | **Boy (Language)** | | | **Girl (Language)** | | |
| --- | --- | --- | --- | --- | --- | --- | --- | --- | --- | --- | --- | --- |
| **Characteristic** | **OR** | **95% CI** | **p-value** | **OR** | **95% CI** | **p-value** | **OR** | **95% CI** | **p-value** | **OR** | **95% CI** | **p-value** |
| **Age** | 0.79 | 0.68, 0.92 | 0.002 | 0.76 | 0.65, 0.90 | <0.001 | 0.82 | 0.70, 0.96 | 0.016 | 0.85 | 0.72, 1.01 | 0.064 |
| **Parental education** | reference: low | | | | | | | | | | | |
| medium | 4.05 | 1.33, 12.3 | 0.014 | 1.49 | 0.67, 3.31 | 0.3 | 1.86 | 0.71, 4.89 | 0.2 | 1.32 | 0.59, 2.98 | 0.5 |
| high | 6.11 | 1.97, 18.9 | 0.002 | 2.01 | 0.88, 4.62 | 0.10 | 2.61 | 0.96, 7.05 | 0.059 | 2.08 | 0.89, 4.85 | 0.089 |
| **Family income** | reference: low/medium | | | | | | | | | | | |
| medium | 1.33 | 0.93, 1.92 | 0.12 | 1.21 | 0.83, 1.76 | 0.3 | 1.40 | 0.96, 2.05 | 0.083 | 1.44 | 0.96, 2.14 | 0.077 |
| medium/high | 2.70 | 1.57, 4.64 | <0.001 | 1.13 | 0.68, 1.89 | 0.6 | 2.31 | 1.30, 4.09 | 0.004 | 1.56 | 0.89, 2.75 | 0.12 |
| high | 2.17 | 1.35, 3.49 | 0.001 | 2.21 | 1.39, 3.52 | <0.001 | 2.54 | 1.51, 4.25 | <0.001 | 1.75 | 1.05, 2.91 | 0.032 |
| **HRQoL (overall)** | 1.07 | 1.04, 1.10 | <0.001 | 1.06 | 1.03, 1.09 | <0.001 | 1.04 | 1.01, 1.07 | 0.014 | 1.03 | 1.00, 1.06 | 0.036 |
| **Stressful life events** | 1.07 | 1.01, 1.14 | 0.030 | 0.96 | 0.90, 1.02 | 0.2 | 1.00 | 0.94, 1.07 | >0.9 | 0.99 | 0.93, 1.06 | 0.9 |
| **BMI z-score** | 0.90 | 0.79, 1.03 | 0.12 | 0.94 | 0.82, 1.08 | 0.4 | 0.96 | 0.84, 1.11 | 0.6 | 0.78 | 0.67, 0.91 | 0.001 |
| **Healthy diet score** | 1.00 | 0.98, 1.02 | 0.8 | 1.00 | 0.98, 1.02 | 0.7 | 1.01 | 0.99, 1.04 | 0.2 | 1.03 | 1.01, 1.05 | 0.005 |
| **Weekly media use (hours)** | 0.98 | 0.97, 1.00 | 0.012 | 0.99 | 0.97, 1.00 | 0.046 | 0.97 | 0.96, 0.99 | <0.001 | 0.97 | 0.96, 0.99 | <0.001 |
| **Sports club (hours)** | 1.08 | 1.03, 1.13 | <0.001 | 1.12 | 1.06, 1.18 | <0.001 | 1.05 | 1.01, 1.11 | 0.030 | 1.08 | 1.02, 1.14 | 0.009 |
| **Average nightly sleep duration (hours)** | 1.08 | 0.93, 1.26 | 0.3 | 0.88 | 0.75, 1.01 | 0.076 | 1.08 | 0.92, 1.26 | 0.4 | 0.88 | 0.76, 1.04 | 0.13 |
| **Sleep quality** | reference: good | | | | | | | | | | | |
| bad | 0.74 | 0.42, 1.29 | 0.3 | 0.85 | 0.53, 1.37 | 0.5 | 0.72 | 0.40, 1.29 | 0.3 | 0.57 | 0.34, 0.95 | 0.031 |
| **Variance at the country level** | 1.06 | | | 2.11 | | | 2.65 | | | 3.10 | | |
| **Unconditional ICC** | 0.21 | | | 0.35 | | | 0.40 | | | 0.44 | | |
| **Conditional ICC** | 0.24 | | | 0.39 | | | 0.45 | | | 0.49 | | |
| **Observations (N)** | 1051 | | | 1132 | | | 1050 | | | 1140 | | |

***Note***. All models include random effects at the country level (9 countries in total). Odds ratio (OR) and 95% CI were provided in the table. We reported the results of all pre-planned comparisons with p-values. This is a complete-case analysis, only including participants with all independent variables and at least one academic subject grade available. ICC is intraclass correlation coefficient.

**Table S4**. Fully adjusted multivariable logistic regression models predicting math and language grades by sex groups, with multiple imputation for missing data and country fixed effects.

|  | **Boy (Math)** | | | **Girl (Math)** | | | **Boy (Language)** | | | **Girl (Language)** | | |
| --- | --- | --- | --- | --- | --- | --- | --- | --- | --- | --- | --- | --- |
| **Characteristic** | **OR** | **95% CI** | **p-value** | **OR** | **95% CI** | **p-value** | **OR** | **95% CI** | **p-value** | **OR** | **95% CI** | **p-value** |
| **Age** | 0.74 | 0.66, 0.83 | <0.001 | 0.77 | 0.68, 0.87 | <0.001 | 0.80 | 0.71, 0.91 | <0.001 | 0.90 | 0.79, 1.03 | 0.135 |
| **Parental education** | reference: low | | | | | | | | | | | |
| medium | 2.02 | 1.03, 3.97 | 0.040 | 1.75 | 0.96, 3.21 | 0.069 | 2.11 | 1.01, 4.37 | 0.046 | 1.97 | 1.08, 3.61 | 0.028 |
| high | 2.93 | 1.46, 5.85 | 0.002 | 2.86 | 1.51, 5.41 | 0.001 | 2.84 | 1.34, 6.02 | 0.006 | 3.43 | 1.80, 6.53 | <0.001 |
| **Family income** | reference: low/medium | | | | | | | | | | | |
| medium | 1.27 | 0.92, 1.75 | 0.141 | 1.29 | 0.93, 1.78 | 0.127 | 1.24 | 0.89, 1.73 | 0.201 | 1.44 | 1.02, 2.03 | 0.040 |
| medium/high | 2.11 | 1.30, 3.45 | 0.003 | 1.09 | 0.70, 1.68 | 0.708 | 2.05 | 1.25, 3.36 | 0.005 | 1.43 | 0.87, 2.35 | 0.155 |
| high | 1.80 | 1.19, 2.72 | 0.005 | 1.90 | 1.27, 2.85 | 0.002 | 2.15 | 1.42, 3.26 | <0.001 | 1.76 | 1.14, 2.73 | 0.011 |
| **HRQoL (overall)** | 1.05 | 1.03, 1.08 | <0.001 | 1.05 | 1.02, 1.07 | <0.001 | 1.05 | 1.02, 1.08 | <0.001 | 1.03 | 1.01, 1.06 | 0.007 |
| **Stressful life events** | 1.06 | 1.01, 1.12 | 0.017 | 0.98 | 0.94, 1.03 | 0.494 | 0.99 | 0.94, 1.05 | 0.788 | 0.98 | 0.93, 1.04 | 0.516 |
| **BMI z-score** | 0.90 | 0.81, 1.00 | 0.057 | 0.93 | 0.83, 1.04 | 0.182 | 0.91 | 0.81, 1.01 | 0.085 | 0.79 | 0.70, 0.89 | <0.001 |
| **Healthy diet score** | 1.01 | 0.99, 1.03 | 0.220 | 1.01 | 0.99, 1.02 | 0.399 | 1.02 | 0.998, 1.03 | 0.082 | 1.03 | 1.01, 1.04 | 0.004 |
| **Weekly media use (hours)** | 0.99 | 0.98, 1.00 | 0.186 | 0.99 | 0.98, 0.997 | 0.016 | 0.98 | 0.97, 0.99 | <0.001 | 0.97 | 0.96, 0.99 | <0.001 |
| **Sports club (hours)** | 1.07 | 1.03, 1.11 | 0.001 | 1.10 | 1.05, 1.15 | <0.001 | 1.06 | 1.01, 1.10 | 0.009 | 1.07 | 1.02, 1.12 | 0.008 |
| **Average nightly sleep duration (hours)** | 1.04 | 0.93, 1.17 | 0.488 | 0.95 | 0.84, 1.08 | 0.412 | 1.06 | 0.93, 1.20 | 0.399 | 0.92 | 0.81, 1.05 | 0.203 |
| **Sleep quality** | reference: good | | | | | | | | | | | |
| bad | 0.84 | 0.55, 1.30 | 0.440 | 0.98 | 0.67, 1.44 | 0.920 | 0.95 | 0.60, 1.51 | 0.829 | 0.65 | 0.43, 0.98 | 0.040 |

***Note***. All models include country fixed effects (9 countries, represented by 8 dummy variables), but these are omitted from the table as they are not of primary interest. The table presents odds ratios (OR) with 95% confidence intervals (CI). All pre-planned comparisons are reported with p-values. Multiple imputation was used for missing data in the predictors (20 imputed datasets).

**Table S5**. Fully adjusted multivariable logistic regression models predicting poor academic performance (coded as 1) in math and language grades by sex groups. Poor performance is defined using a reverse dichotomization, where “Pass or sufficient” and “Fail or insufficient” are classified as poor, compared to higher grades.

|  | **Boy (Math)** | | | **Girl (Math)** | | | **Boy (Language)** | | | **Girl (Language)** | | |
| --- | --- | --- | --- | --- | --- | --- | --- | --- | --- | --- | --- | --- |
| **Characteristic** | **OR** | **95% CI** | **p-value** | **OR** | **95% CI** | **p-value** | **OR** | **95% CI** | **p-value** | **OR** | **95% CI** | **p-value** |
| **Age** | 1.05 | 0.87, 1.25 | 0.6 | 1.41 | 1.16, 1.72 | <0.001 | 1.26 | 1.05, 1.52 | 0.013 | 1.39 | 1.12, 1.73 | 0.003 |
| **Parental education** | reference: low | | | | | | | | | | | |
| medium | 0.29 | 0.15, 0.58 | <0.001 | 0.52 | 0.26, 1.06 | 0.068 | 0.57 | 0.28, 1.16 | 0.12 | 0.74 | 0.34, 1.69 | 0.5 |
| high | 0.24 | 0.11, 0.50 | <0.001 | 0.32 | 0.14, 0.71 | 0.004 | 0.38 | 0.18, 0.83 | 0.014 | 0.67 | 0.28, 1.65 | 0.4 |
| **Family income** | reference: low/medium | | | | | | | | | | | |
| medium | 0.86 | 0.57, 1.29 | 0.5 | 0.75 | 0.47, 1.20 | 0.2 | 0.61 | 0.39, 0.94 | 0.025 | 0.85 | 0.51, 1.44 | 0.6 |
| medium/high | 0.30 | 0.13, 0.63 | 0.003 | 0.48 | 0.22, 1.02 | 0.064 | 0.50 | 0.24, 1.00 | 0.056 | 0.31 | 0.11, 0.77 | 0.016 |
| high | 0.44 | 0.23, 0.82 | 0.012 | 0.62 | 0.32, 1.19 | 0.2 | 0.39 | 0.19, 0.76 | 0.007 | 0.43 | 0.19, 0.93 | 0.039 |
| **HRQoL (overall)** | 0.95 | 0.92, 0.98 | <0.001 | 0.98 | 0.94, 1.01 | 0.12 | 0.95 | 0.92, 0.98 | 0.003 | 0.99 | 0.95, 1.03 | 0.5 |
| **Stressful life events** | 0.95 | 0.88, 1.03 | 0.2 | 1.04 | 0.96, 1.12 | 0.3 | 1.01 | 0.93, 1.09 | 0.8 | 1.06 | 0.97, 1.16 | 0.2 |
| **BMI z-score** | 1.07 | 0.92, 1.25 | 0.4 | 0.97 | 0.81, 1.16 | 0.7 | 1.15 | 0.98, 1.35 | 0.10 | 1.17 | 0.95, 1.45 | 0.14 |
| **Healthy diet score** | 1.00 | 0.98, 1.03 | 0.9 | 0.99 | 0.97, 1.02 | 0.6 | 0.96 | 0.94, 0.99 | 0.004 | 0.97 | 0.94, 1.00 | 0.059 |
| **Weekly media use (hours)** | 1.01 | 1.00, 1.03 | 0.064 | 1.02 | 1.01, 1.04 | 0.005 | 1.01 | 0.99, 1.02 | 0.2 | 1.03 | 1.01, 1.05 | 0.006 |
| **Sports club (hours)** | 0.91 | 0.85, 0.97 | 0.006 | 0.80 | 0.72, 0.88 | <0.001 | 0.93 | 0.87, 0.99 | 0.034 | 0.90 | 0.81, 0.99 | 0.036 |
| **Average nightly sleep duration (hours)** | 0.87 | 0.73, 1.03 | 0.10 | 1.12 | 0.93, 1.34 | 0.2 | 0.87 | 0.73, 1.04 | 0.12 | 1.08 | 0.88, 1.32 | 0.4 |
| **Sleep quality** | reference: good | | | | | | | | | | | |
| bad | 0.74 | 0.37, 1.42 | 0.4 | 1.31 | 0.71, 2.35 | 0.4 | 0.91 | 0.45, 1.76 | 0.8 | 0.97 | 0.48, 1.88 | >0.9 |
| **Observations (N)** | 1,051 | | | 1,132 | | | 1,050 | | | 1,140 | | |

***Note***. All models include country fixed effects (9 countries, represented by 8 dummy variables), but these are omitted from the table as they are not of primary interest. Odds ratio (OR) and 95% CI were provided in the table. We reported the results of all pre-planned comparisons with p-values. This is a complete-case analysis, only including participants with all independent variables and at least one academic subject grade available.

**Figure S1.** Proportion of high achievers (who achieved the highest two levels of Excellent and Good) in math and language by sex groups.

**
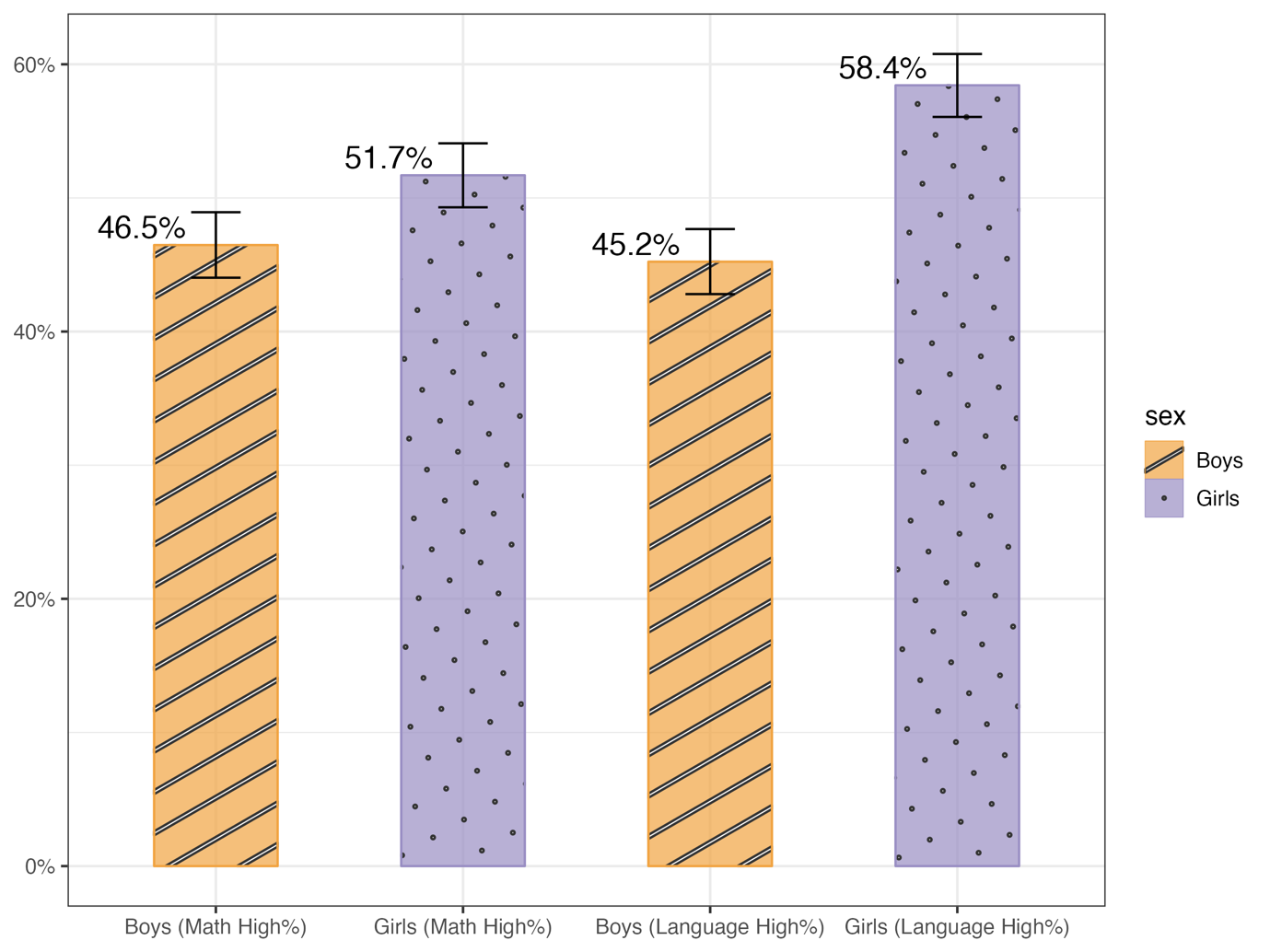
**

***Note***: exact (Clopper-Pearson) confidence intervals for proportions were presented in the Figure.

**Figure S2**. Forest plots for key indicators of interest in the fully adjusted multivariable logistic regression models predicting math and language grades by sex groups.

***
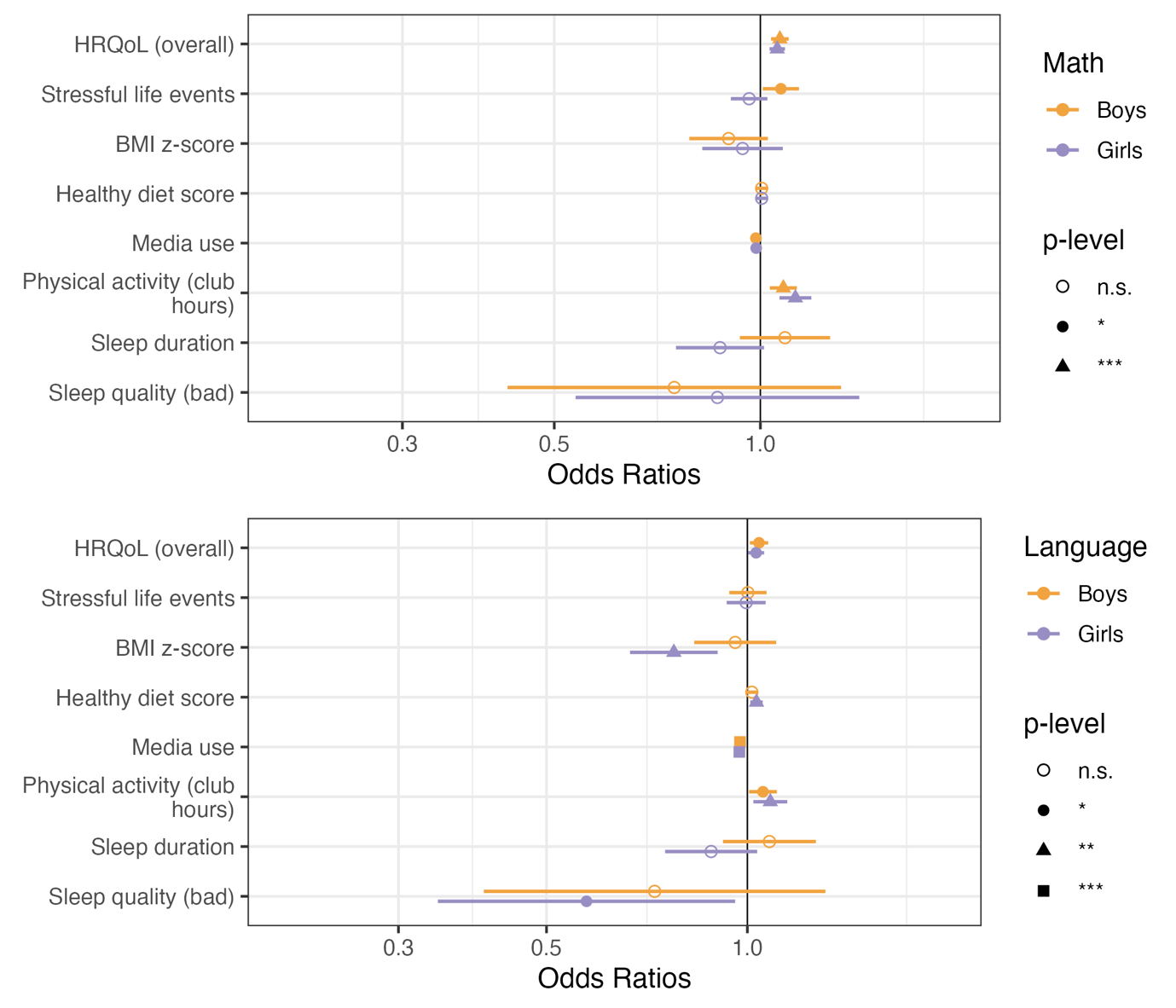
Note***. *** p < 0.001, ** p < 0.01, * p < 0.05, n.s. indicates not significant at 5% level.

**List of the 12 questions for HRQoL**

- Emotional well-being
  - I had fun and laughed a lot
  - I was bored
  - I felt alone
  - I felt scared or unsure of myself
- Self -esteem
  - I was proud of myself
  - I felt on top of the world
  - I felt pleased with myself
  - I had lots of good ideas
- Family
  - I got on well with my parents
  - I felt fine at home
  - We quarreled at home
  - I felt restricted by my parents
- Social context
  - I did things together with my friends
  - I was a “success” with my friends
  - I got along well with my friends
  - I felt different from other people

**List of stressful life events**

1. Divorce of separation of parents/step-parents
2. Death of a parent/step-parent
3. Death of a sibling
4. Death of a grandparent or other family member
5. Addition of new family member (e.g., step-parent or birth of a sibling)
6. Job loss of mother and/or father
7. Major frustrations at school (e.g., with teachers, year repetition, bad marks, school fail)
8. Major frustrations with peers
9. Long-term separation from a close family member (e.g., parent starts working abroad, sibling moved out)
10. Serious disease, surgery, or accidents
11. Serious disease, surgery, or accidents of a family member
12. Moving (into a new flat/family home)
13. Other important events
